# Supplementary material for: Three-Dimensional Hepatocyte Spheroids: Model for Assessing Chemotherapy in Hepatocellular Carcinoma
Source: Biomedicines. 2024 May 28;12(6):1200. doi: 10.3390/biomedicines12061200 (PMC11201042; doi:10.3390/biomedicines12061200)
Supplement: Supplementary file 1 [file biomedicines-12-01200-s001.zip › biomedicines-2896714-supplementary.pdf]

biomedicines

MDPI

Three-Dimensional Hepatocyte Spheroids: Model for Assessing Chemotherapy in Hepatocellular Carcinoma

Supplemental Figures

Figure S1; Characterization of EVs preparations isolated by ultracentrifugation from the culture media of TMCRE spheroids.

Figure S2; Tracking of fluorescent EVs by flow cytometry of cultured media.

Figure S3; Characterization of mixed spheroids by histology.

Figure S4; Average size of mixed spheroids.

Figure S5; Gatting of the tumoral cells and hepatocytes.

Figure S6; Changes in the concentration of fluorescent vesicles in the extracellular media of spheroids treated with cytotoxic drugs, according to the cytometer analysis.

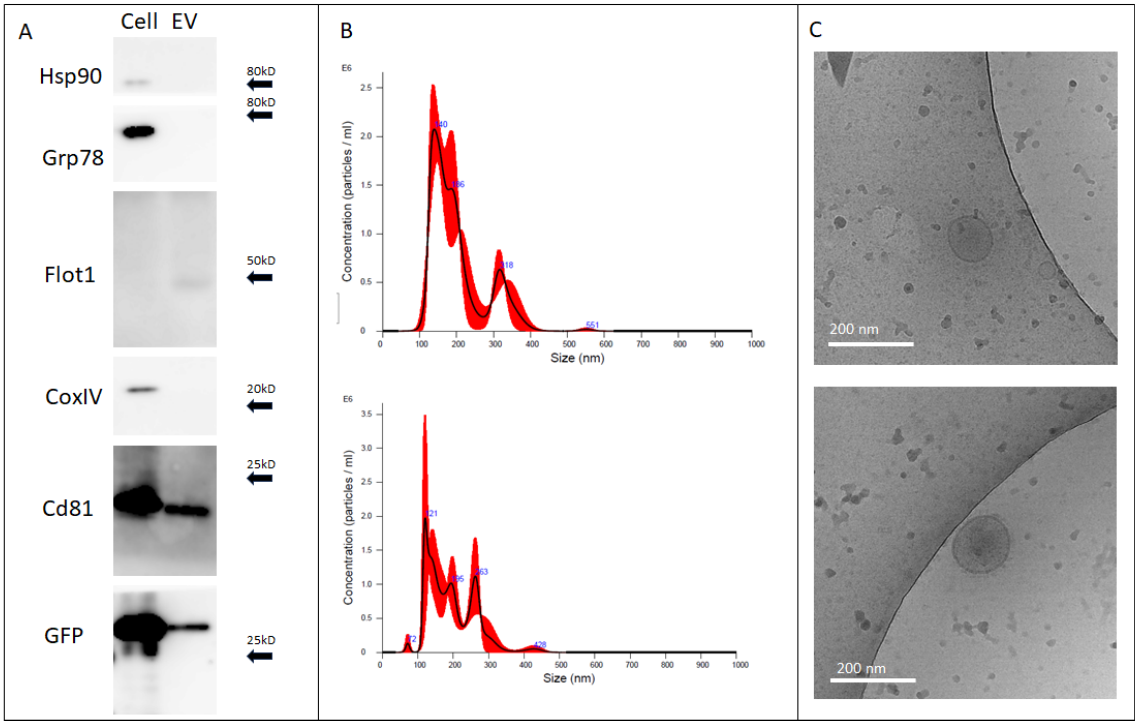

Supplemental Figure S1. Characterization of EVs preparations isolated by ultracentrifugation from the culture media of TMCRE spheroids. **A**, In the panel we show the presence of vesicle markers such CD81 and Flot1, while some cellular marker such HSP90 or GRP78 are absent in the EVs ultracentrifuged preparations. The membrane marker tagged with GFP is present in the EV preparation. **B** The size profile of two independent EV preparations according to NTA analysis. **C** Cryo Electron microscopy of vesicles presented in the preparations.

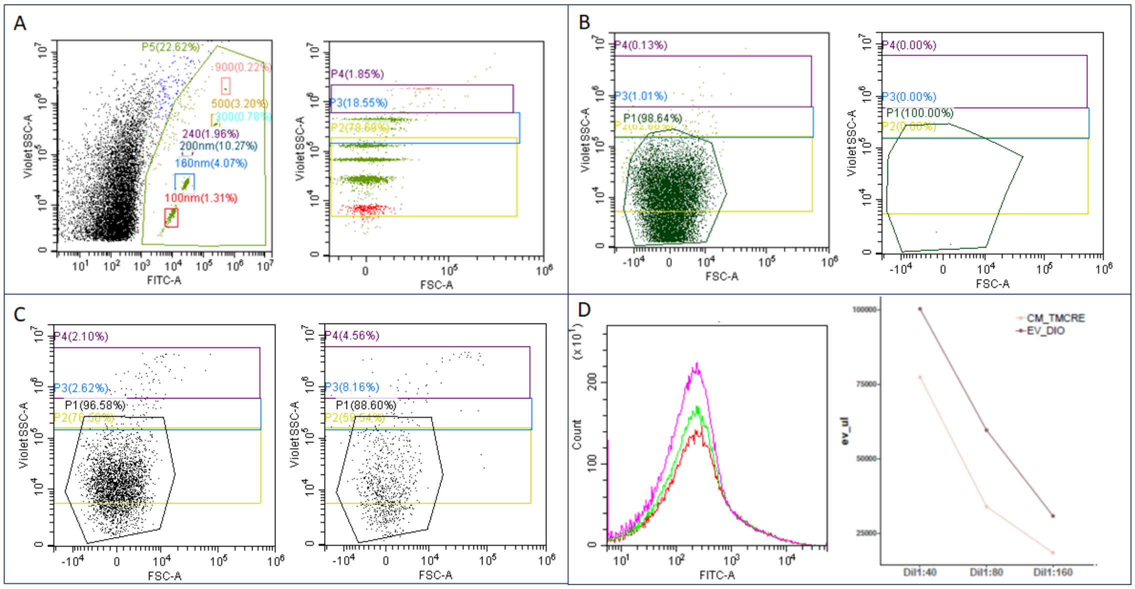

Supplemental Figure S2. Tracking of fluorescent EVs by flow cytometry of cultured media. **A** Gating by fluorescence of a mix of fluorescent polystyrene beads (megamix) of known sizes. **B** In the left, we gate the fluorescent events observed in a EVs isolated by ultracentrifugation from culture media of green fluorescent labelled cells. In the absence of fluorescence, no events were observed (right). **C** Events observed in culture media of spheroids formed with TMCRE hepatocytes (left). The number of events was reduced by a 70 % when media is treated with 0.1% Tx-100 detergent for 5 min. **D** Serial dilution of the conditioned media (CM_TMCRE) decreases the concentration of events present. This linearity effect can be observed also in a preparation of EVs obtained by ultracentrifugation (EV_DIO).

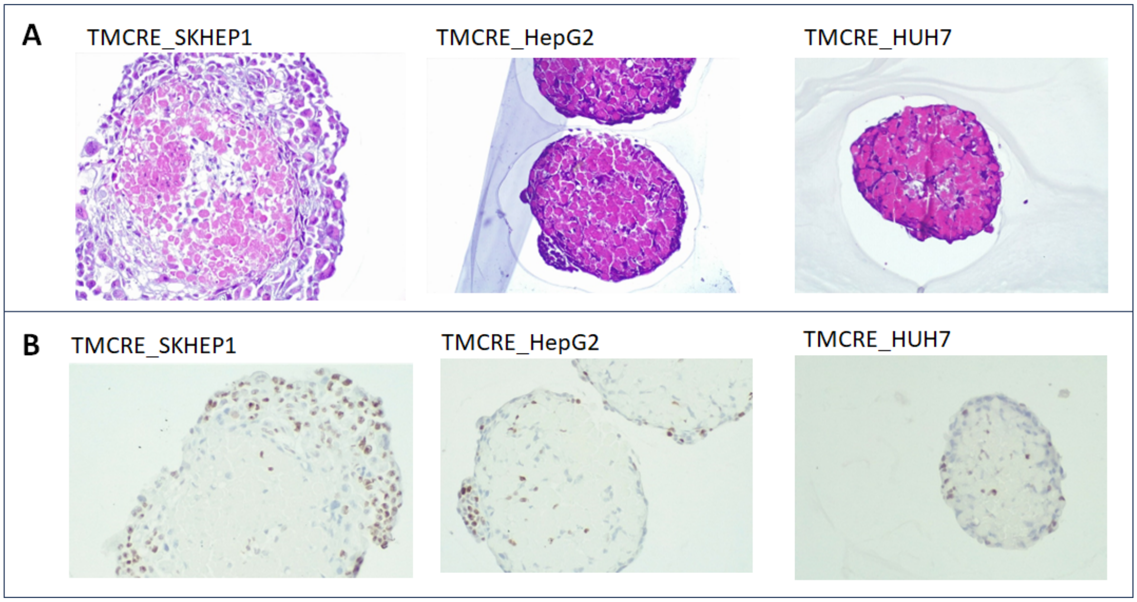

Supplemental Figure S3. Characterization of mixed spheroids by histology. **A**. Hematoxilin eosine staining of a section of 7 days spheroids. **B**. Immunohistochemistry against Ki67 protein.

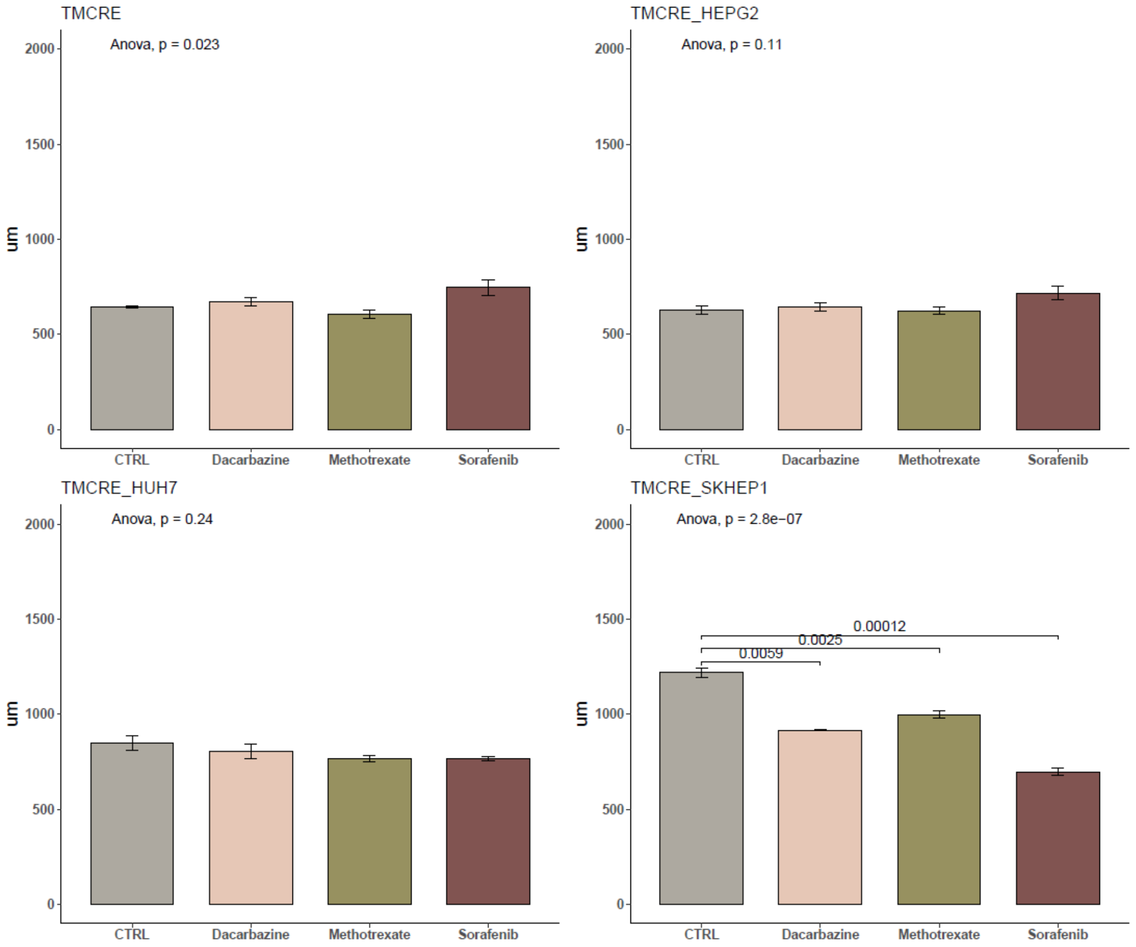

Supplemental Figure S4. Average size of mixed spheroids. Measurement of the diameter of the co-culture of hepatocytes with different tumor cell lines, on day 14, after cytotoxic drug treatments. ANOVA was performed in all the groups, and only planned contrast between control and treatment were performed. We only show significant results (n=3)

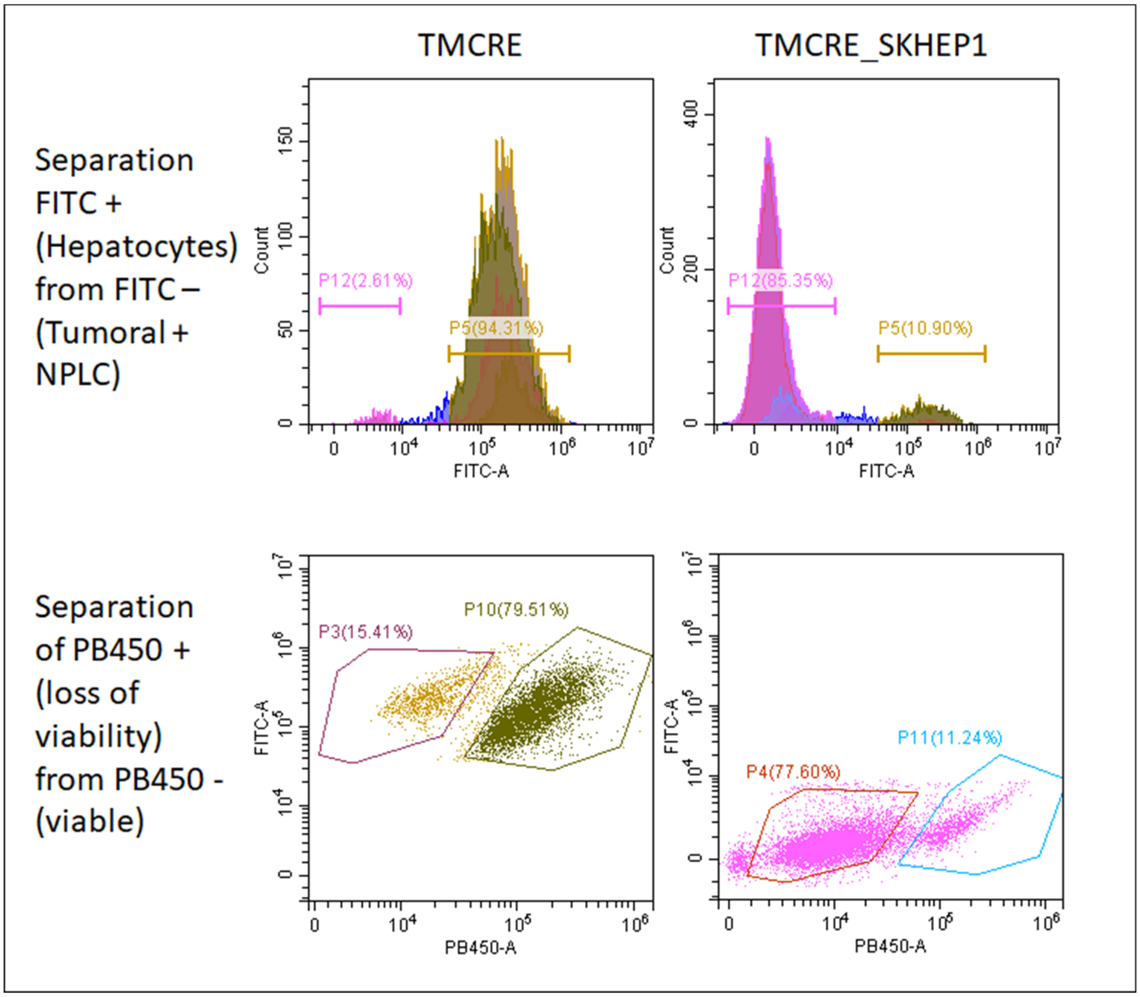

Supplemental Figure S5. Gatting of the tumoral cells and hepatocytes. The gating was performed using the fluorescence in the green channel. Afterwards, we separate vital from damaged cells by the fluorescence on the pacific blue channel, regarding the permeability to a vitality reporter. The examples correspond to hepatocyte spheroids, and to the mix spheroids with the tumoral cell SK-HEP1.

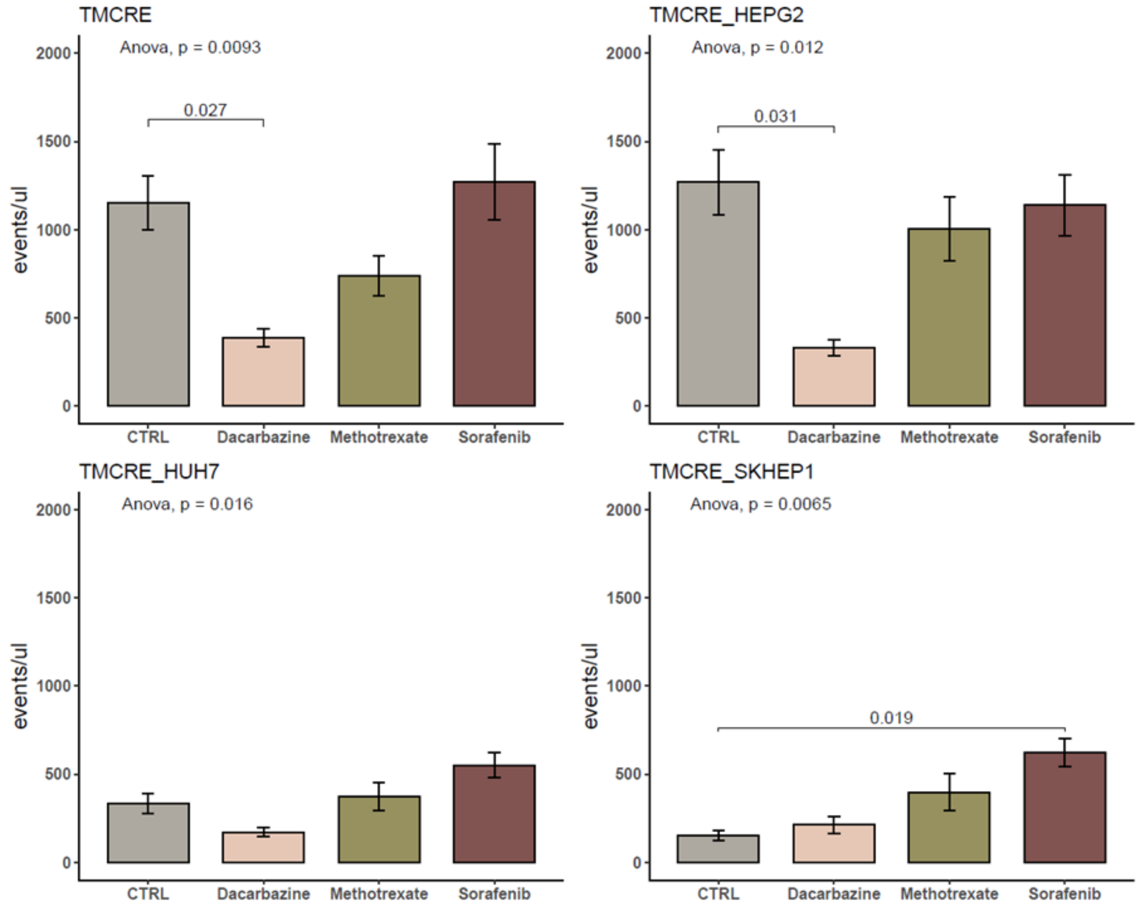

Supplemental Figure S6. Changes in the concentration of fluorescent vesicles in the extracellular media of spheroids treated with cytotoxic drugs, according to the cytometer analysis. ANOVA comparisons were performed among all groups, and planned contrast of control vs each treatment was performed by *t*-test, and only statistically significant results are shown (n=3).
